# In Vitro Alteration by Dentine and Protein of the Antimicrobial Activity of Two Endodontic Irrigants: HybenX^®^ and Sodium Hypochlorite

**DOI:** 10.3390/antibiotics9110792

**Published:** 2020-11-10

**Authors:** Riccardo Pace, Fabio Morecchiato, Luca Giovannini, Luca Di Nasso, Valentina Giuliani, Debora Franceschi, Gabriella Pagavino, Gian Maria Rossolini, Alberto Antonelli

**Affiliations:** 1 Odontostomatology-Endodontic Unit, Careggi University Hospital, 50134 Florence, Italy; riccardo.pace@unifi.it (R.P.); luca.dinasso@gmail.com (L.D.N.); dr.v.giuliani@gmail.com (V.G.); debora.franceschi@unifi.it (D.F.); 2 Department of Experimental and Clinical Medicine, University of Florence, 50134 Florence, Italy; fabio.morecchiato@unifi.it (F.M.); lucagiova3@gmail.com (L.G.); gabriella.pagavino@unifi.it (G.P.); gianmaria.rossolini@unifi.it (G.M.R.); 3 Clinical Microbiology and Virology Unit, Careggi University Hospital, 50134 Florence, Italy

**Keywords:** irrigants, endodontic infections, dentine, *Enterococcus faecalis*

## Abstract

Irrigant solutions commonly used for the treatment of endodontic infections can be inhibited by both organic and inorganic substances. The aim of this study was to evaluate the in vitro antimicrobial activity of the novel irrigant HybenX^®^ and 2.5% and 5% sodium hypochlorite against *Enterococcus faecalis*, in presence of dentine powder (DP) or bovine serum albumin 20% (BSA) as inhibitory agents. An *E. faecalis* American Type Culture Collection (ATCC) 29212 suspension was added to the irrigants (Hybenx^®^ or NaOCl) and one or two different inhibitors (BSA and DP) either after one-hour pre-incubation at 35 ± 1 °C or not. The antimicrobial activity of HybenX^®^ against *E. faecalis* was already proved at 15 min and was neither affected by BSA nor by DP or combinations thereof. NaOCl 2.5% showed an effective antimicrobial activity starting from 15 min and this activity was partially inhibited by BSA and BSA plus DP combination within one hour when pre-incubation occurred. NaOCl 5% showed antimicrobial activity within 15 min, which was inhibited within one hour only in the presence of both BSA and DP regardless of the pre-incubation period. HybenX^®^ could represent a good alternative to common irrigants for the treatment of *E. faecalis* endodontic infections, showing a rapid antimicrobial activity not inhibited by organic and inorganic inhibitors.

## 1. Introduction

The success of endodontic treatment is strongly influenced by the control of residual intracanal infections and the prevention of reinfections [1,2,3]. For this reason, irrigant solutions with bactericidal activity are used during endodontic treatment. In failed endodontic treatments, *Enterococcus faecalis* is the dominant species recovered [4] and can remain viable in root-filled canals up to 12 months after therapy [5].

Irrigant solutions used in the endodontic treatment may exhibit an excellent bactericidal activity when used in vitro, with rapid bacterial killing also at low concentrations. In vivo, however, many factors, such as the presence of organic compounds (e.g., albumin) present in inflammatory exudate and hydroxylapatite (main inorganic component of dentine) can reduce the antibacterial efficacy of irrigant solutions [6]. Furthermore, the co-administration of different irrigant solutions can show limited benefits [7].

Sodium hypochlorite (NaOCl) is among the most commonly used canal irrigants, due to its strong bactericidal activity [8,9]. However, the antimicrobial activity of sodium hypochlorite is decreased in the presence of dentine and organic compounds [10]. In fact, several studies demonstrated that NaOCl significantly reduced the bacterial load of *E. faecalis*, but is not able to completely eradicate the infection [11,12,13,14].

Hence, the interest for irrigant solutions with improved antibacterial properties for effective root canal disinfection has been increasing [15]. HybenX^®^ (EPIEN Medical, Saint Paul, MN, USA) is a new irrigant preparation, based on a mixture of acidified phenolics, that has been developed as an alternative to conventional irrigants. Recent studies have demonstrated the effectiveness of this preparation in the treatment of clinical cases showing acute periodontal abscess without the use of systemic or local antibiotics [16,17]. Similar favorable outcomes were obtained in the treatment of peri-implant mucositis and peri-implantitis [18,19,20]. In addition, a randomized controlled trial demonstrated the beneficial effects of this preparation for the treatment of oral aphthae [21]. Indeed, in a recent study, HybenX^®^ showed notable antimicrobial activity in terms of minimum inhibitory, bactericidal and fungicidal concentrations against the reference strains of several bacterial and fungal species [22], while no data are currently available about the in vitro activity of HybenX^®^ on other endodontic pathogens such as streptococci and no information is available on the potential inhibitory activity by dentine and organic compounds.

The aim of the present study was to evaluate the in vitro antimicrobial effect of HybenX^®^ and sodium hypochlorite against *Enterococcus faecalis*, in the presence of dentine powder (DP) or bovine serum albumin (BSA) as potential inhibitory agents encountered in vivo.

## 2. Results

Bactericidal activity of HybenX^®^ and of two different NaOCl stock solutions (2.5% and 5%) against the reference strain of *E. faecalis* ATCC 29212 was investigated either in the absence or presence of DP (56% *w*/*v* stock) and/or BSA (20% *w*/*v* stock) as potential inhibitors.

The presence of DP and/or BSA did not affect *E. faecalis* viability, and survival curves in their presence were comparable with that observed in normal saline (data not shown).

**Figure 1:**
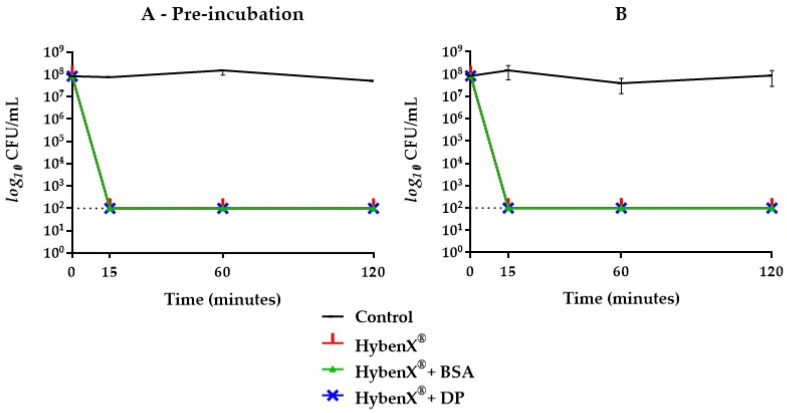
*E. faecalis* ATCC 29212 survival curves in HybenX^®^ groups with (**A**) and without (**B**) one-hour pre-incubation in combination with bovine serum albumin (BSA) or dentine powder (DP). Final concentrations were one fourth of the original. The dotted line indicates the limit of detection (100 colony forming units (CFU)/mL).

**Figure 2:**
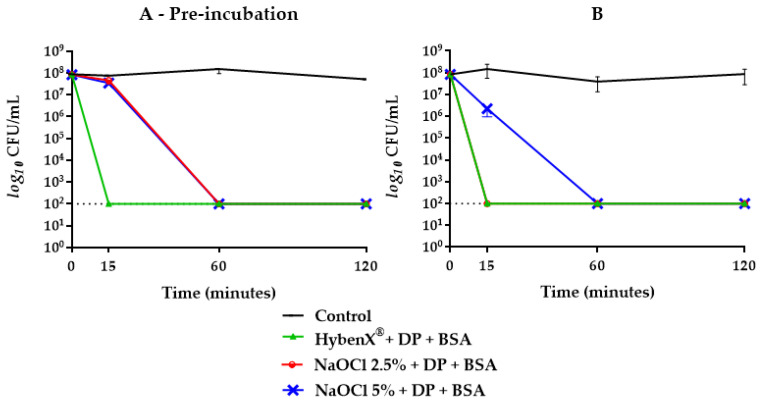
*E. faecalis* ATCC 29212 survival curves in HybenX^®^ and NaOCl groups tested with DP plus BSA with (**A**) and without (**B**) one-hour pre-incubation. Final concentrations were one sixth of the original. The dotted line indicates the limit of detection (100 CFU/mL).

**Figure 3:**
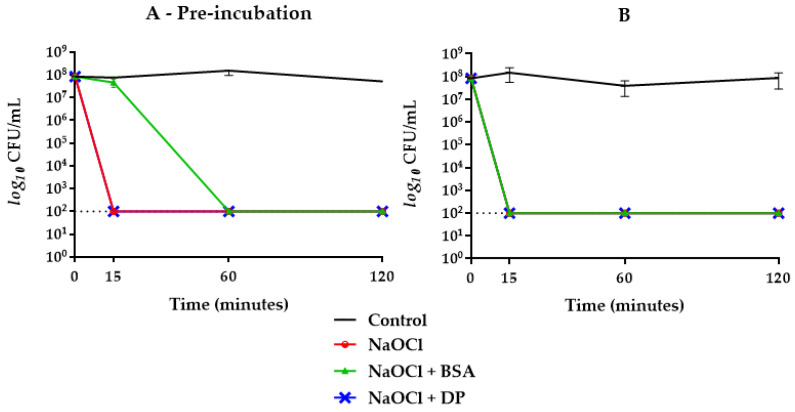
*E. faecalis* ATCC 29212 survival curves in NaOCl 2.5% groups with (**A**) and without (**B**) one-hour pre-incubation in combination with BSA or DP. Final concentrations were one fourth of the original. The dotted line indicates the limit of detection (100 CFU/mL).

HybenX^®^ showed a strong bactericidal activity against *E. faecalis*, with a reduction of the initial inoculum below the limit of detection (>5 logs) already observed after 15 min of incubation. The presence of BSA or DP had no inhibitory effect on the bactericidal activity of HybenX^®^, even after a pre-incubation step with the potential inhibitors (Figure 1). Even when the concentration of HybenX^®^ was further reduced (from one fourth to one sixth of the original) and both potential inhibitors added together, no reduction of its bactericidal activity was achieved regardless of the incubation step (Figure 2). NaOCl, either at 2.5% or 5%, showed a strong bactericidal activity against *E. faecalis*, with a reduction of the initial inoculum below the limit of detection (>5 logs) already observed after 15 min of incubation. The bactericidal activity of NaOCl, however, was affected by the presence of BSA alone when the pre-incubation step occurred (Figure 3).

**Figure 4:**
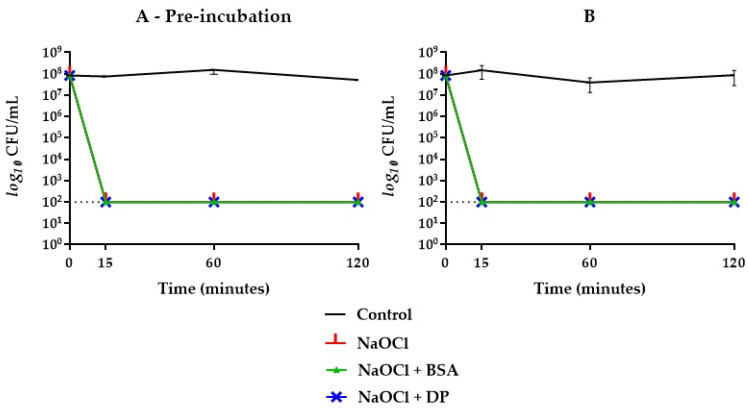
*E. faecalis* ATCC 29212 survival curves in NaOCl 5% groups with (**A**) and without (**B**) one-hour pre-incubation in combination with BSA or DP. Final concentrations were one fourth of the original. The dotted line indicates the limit of detection (100 CFU/mL).

When BSA was combined with DP, the bactericidal activity of NaOCl 2.5% was delayed up to 60 min when subjected to prior incubation, while without pre-incubation, no inhibitory effect was achieved (Figure 2). Concerning NaOCl 5%, neither BSA or DP alone inhibited its bactericidal activity (Figure 4). By combining BSA and DP, an inhibitory effect was revealed in the first 15 min, with a slightly higher effect when not subjected to a prior incubation step (Figure 2).

## 3. Discussion

In this study, HybenX^®^ showed a strong bactericidal activity against *E. faecalis* ATCC 29212 (reduction of the bacterial inoculum by >5 logs within 15 min), comparable to that achieved with NaOCl which is the conventional agent used as irrigant during endodontic treatment.

The bactericidal activity of HybenX^®^ even at low concentration, however, was not affected by the presence of DP or BSA as potential inhibitors, while this was not the case for NaOCl, which, at a lower concentration, was inhibited by both BSA and BSA plus DP, while at higher concentration only BSA plus DP showed an inhibitory effect on its bactericidal activity. The inhibitory activity of BSA on the bactericidal activity of NaOCl has been already reported by Pappen et al. [23], while no data regarding the inhibition of the bactericidal activity of HybenX^®^ was previously reported.

The reason by which the bactericidal activity of HybenX^®^ was not affected by BSA remains to be clarified. HybenX^®^ has a strong affinity for water: the sulphate group in relation to oxygen atoms has a strong negative charge able to attract water molecules causing the dehydration of the surface. The absence of an inhibitory activity of BSA on HybenX^®^ could be dependent on this chemical feature, or on the possibly higher intrinsic bactericidal activity of the product, which is not easily inhibited in the presence of proteins such as BSA.

Portenier et al. [6] carried out similar research evaluating the potential inhibitory effect of serum albumin and Sassone et al. [24], using BSA 0.5%, did not report inhibitory effects on the bactericidal efficacy of 1% and 5% sodium hypochlorite. Pappen et al. [23] evaluated the inhibitory potential of BSA against higher concentrations of sodium hypochlorite (6.66%) reporting that BSA had a concentration-dependent inhibitory action on the antibacterial activity of NaOCl.

In this study, sodium hypochlorite, at the concentrations of 2.5% and 5%, expressed a rapid and effective antibacterial activity against *E. faecalis* ATCC 29212 as well as HybenX^®^. In groups where BSA alone and in combination with DP was present, the inhibitory activity changed depending on the concentration of hypochlorite used (Figure 2, Figure 3 and Figure 4). NaOCl at 5% was not affected by the action of BSA alone (Figure 4), while the antimicrobial activity against *E. faecalis* ATCC 29212 was partially inhibited within the first hour by the simultaneous presence of DP and BSA regardless of the incubation (Figure 2).

On the other hand, BSA alone exerted an inhibitory effect only on NaOCl 2.5% with a greater slowdown on its antimicrobial activity when subjected to one-hour pre-incubation (Figure 3). When BSA was combined with DP, a greater inhibitory activity was shown in pre-incubated samples with a kinetic similar to that observed with NaOCl 5% (Figure 2).

In this research, NaOCl was not inhibited by DP alone in any case (Figure 2, Figure 3 and Figure 4). These results differ partially from what other researchers have observed. Haapasalo et al. [11] developed a method to produce dentine powder to perform in vitro research in a standardized way and tested the influence of dentine on drugs or root canal medicaments, highlighting an inhibiting effect of bactericidal action. Vianna et al. [25] and Radcliffe et al. [26] observed in vitro that sodium hypochlorite solutions at concentrations of 0.5% and 1%, without the addition of inhibitory substances, still required up to 30 min to kill *E. faecalis* cultures. These contradictory results have been explained by Pappen et al. [23]: it is possible that the organic load by the culture medium contributed to the inhibition of the antimicrobial activity of NaOCl in those studies.

Results presented in this study are similar to those presented by Morgental et al. [27]: using a higher concentration of NaOCl (6%), a low percentage of surviving *E. faecalis* ATCC 29212 was achieved regardless of the presence of DP and the inhibitory activity of DP on NaOCl (1%) was drastically influenced by the solution volume: the higher the volume, the lower the inhibitory activity observed [23,27].

In this study, DP alone showed no inhibitory activity against NaOCl at different concentrations and the influence of the organic material present in the root canal plays a key role in reducing the antibacterial activity of sodium hypochlorite. It has been demonstrated that dentine has a significantly lower inhibitory activity than human serum albumin [28], even at high concentrations [29]. The inhibitory effect of DP on sodium hypochlorite is enhanced when human serum is added. The intensity of this inhibition depends on the drug concentration, the drug-dentine time of contact and the time of exposure of the bacteria to the mixture. By increasing the medicament concentration and the incubation time, the inhibitory activity of the dentine decreases. The use of DP at high concentrations in in vitro experiments hardly simulates the in vivo conditions. In addition, the finely ground DP has a much larger surface area than root canal walls, which increases potential interactions. Lower DP concentrations are likely to better reflect clinical reality [30,31].

Since the bactericidal activity was in most cases higher than 5 logs of the CFU count and bactericidal activity is mainly defined as a reduction of at least 3 logs [32], no further statistical analysis was performed.

## 4. Materials and Methods

### 4.1. Medicaments

The medicaments tested were HybenX^®^ (EPIEN Medical, Saint Paul, MN, USA) and sodium hypochlorite (NaOCl) 5% (©2018 OGNA Lab S.r.l., Muggiò, MB, Italy) and 2.5% (prepared from the 5% solution, by dilution in sterile distilled water (dH_2_O)). HybenX^®^ is a novel hygroscopic solution composed of a mixture of acidified phenolics, consisting of 60% sulfonated phenolics, 28% sulfuric acid, and 12% water. The product is currently marketed by the producer both for use in periodontics (HybenX^®^ Oral Tissue Decontaminant, used in this study) and for endodontics (HybenX^®^ Root Canal Cleanser). The two forms of the product, which have the same chemical composition, differ in consistency: more viscous gel for periodontal use and more liquid gel for endodontic use.

### 4.2. Bacterial Strain

*E. faecalis* ATCC 29212 was used as a test organism and was grown on Mueller-Hinton agar plates (Becton Dickinson and Company, Franklin Lakes, NJ, USA).

### 4.3. Dentine Powder (DP) and Bovine Serum Albumin (BSA)

For the preparation of DP, intact premolars extracted for orthodontic reasons and stored in phosphate buffered saline (PBS) at 4 °C were used. Teeth were cut at the cement–enamel junction, and root canals were prepared using 25# Protaper Gold (Dentsply-Maillefer) to remove pulp tissue and the canal was irrigated with 5% NaOCl and 17% EDTA to remove all traces of organic material and smear layer. The dentine powder was obtained by grinding with a diamond bur, obtaining granules of size <190 μm. DP was sterilized at 121 °C for 15 min and stored at 4 °C until use. Dentine powder was suspended in sterile dH_2_O to a concentration of 56% *w*/*v*.

BSA (Sigma-Aldrich, St. Louis, MO, USA) powder was diluted in sterile dH_2_O in order to obtain a 20% *w*/*v* stock solution.

### 4.4. Workflow

Fifty microliters (50 μL) of BSA or DP suspensions were thoroughly mixed with 50 μL of HybenX^®^ or NaOCl in sterile polypropylene tubes before adding 100 μL of bacterial suspension at 0.5 McFarland (~1.5 × 10^8^ CFU/mL) in NaCl 0.9% for a total volume of 200 µL. The final concentrations were one fourth of the original. *E. faecalis* ATCC 29212 was grown on a Mueller–Hinton agar (MHA) plate and a single colony was picked using a sterile cotton swab, streaked in sterile NaCl 0.9% and measured with a densitometer (Densicheck, bioMèrieux, Marcy l’Etoile, France) up to a 0.5 McFarland density. Control groups consisted of samples where 50 µL of sterile water was used instead of dentine powder or BSA. For groups 7, 11 and 15 (Table 1), a volume of 150 μL of the bacterial suspension was added to 150 μL of the solution containing both putative inhibitors and medicaments, for a final volume of 300 μL to maintain the same bacterial concentration. For these three groups the final concentrations were one sixth of the original.

The inhibitor plus medicament plus bacterial suspensions were carefully mixed for 10 s at the beginning and before each collection. After treatment, a 20 µL aliquot was taken from all suspensions at 15 min, one hour and two hours, then serially 10-fold diluted up to 10^−7^ in sterile NaCl 0.9% and cultured on tryptone soya agar (TSA) (Sigma-Aldrich, St. Louis, MO, USA) plates using spot method (two spots of 5 µL for each test) and incubated at 35 ± 1 °C for 24 h. The plates were inspected for growth, checked for purity, and the colonies were counted (limit of detection 100 CFU/mL).

For each experimental group, a pre-incubation step was also performed: the inhibitor plus medicament solutions were prepared as previously described, then incubated for one hour at 35 ± 1 °C in ambient air before the addition of the bacterial inoculum. The inhibitor plus medicament solutions not subjected to the pre-incubation step were prepared immediately before being processed in order to use the same suspension of *E. faecalis* ATCC 29212 at 0.5 McFarland for each condition.

All conditions were tested in duplicate in three different experiments, mean and standard deviation were calculated and plotted in graphs.

## 5. Conclusions

In the present study, HybenX^®^ showed a significant bactericidal activity against *E. faecalis* ATCC 29212 with extremely rapid action within 15 min (comparable to what was observed with NaOCl at different concentrations), and its effectiveness was not inhibited by the presence of organic (BSA) and/or inorganic (DP) substances even at low concentrations.

Sodium hypochlorite at the used concentrations expressed an effective antibacterial activity against *E. faecalis*, but differences were observed; BSA combined with DP had the ability to significantly delay the killing kinetics of NaOCl 5% within a timeframe of 15 min, in a condition that represents the usual clinical condition. BSA alone showed an inhibitory effect only on NaOCl 2.5% when the pre-incubation step occurred.

For the aforementioned reasons (rapid antimicrobial activity and lack of inhibition by organic and inorganic substances), HybenX^®^ could represent a valid alternative to NaOCl in endodontic settings. Unfortunately, there is still no information on the side effects of treatment with HybenX^®^ compared to NaOCl.

## Figures and Tables

**Table 1:** Experimental groups tested.

**1**	dH_2_O (control)
**2**	dH_2_O + DP
**3**	dH_2_O + BSA
**4**	HybenX^®^ only
**5**	HybenX^®^ + DP
**6**	HybenX^®^ + BSA
**7**	HybenX^®^ + DP + BSA
**8**	NaOCl 2.5%
**9**	NaOCl 2.5% + DP
**10**	NaOCl 2.5% + BSA
**11**	NaOCl 2.5% + DP + BSA
**12**	NaOCl 5%
**13**	NaOCl 5% + DP
**14**	NaOCl 5% + BSA
**15**	NaOCl 5% + DP + BSA
